# Dr. Maynard Gooding Burgess

**Published:** 1914-11

**Authors:** 


Dr. Maynard Gooding Burgess, P. & S. 1898, a veteran of the Spanish-American War, died in Herkimer, August 14, from a self-inflicted gun-shot wound of the head, aged 38. He had been in bad health for some time.

